# Implementing Oncologic Nursing Care Plans in Electronic Health Records With Two Taxonomies: A Pilot Study

**DOI:** 10.1177/01939459241310402

**Published:** 2025-01-06

**Authors:** Serena Togni, Lucia Saracino, Mariangela Cieri, Rosita Bianco, Stefano Terzoni, Santini Magda Giulia, Emanuela Zito, Maura Lusignani, Pazzaglia Maria Silvia, Letizia Depalma

**Affiliations:** 1 Fondazione IRCCS Istituto Nazionale Tumori, Milan, Italy; 2 Department of Biomedical Sciences for Health, University of Milan, Milan, Italy

**Keywords:** electronic health records, nursing care plans, oncology nursing, International Classification for Nursing Practice, Common Terminology Criteria for Adverse Events

## Abstract

**Background:**

Nursing care plans document the nursing process, displaying actions, and illustrating expected outcomes. Their integration into electronic health records (EHRs) is critical for accurate documentation, enhanced by standardized nursing terminologies that promote communication, critical reasoning, and patient safety through consistent language for information.

**Objective:**

This study aimed to identify appropriate standardized nursing terminology tailored to the context of a Northern Italian Cancer Center and research facility for developing nursing care plans and starting their integration into institutional EHRs.

**Methods:**

Participatory action research was conducted to select proper terminology respecting the oncological setting, develop nursing care plans, and start their implementation in EHRs. The nursing team of a pilot ward collaborated closely with the researchers as coresearchers. Care plan samples were presented using the North American Nursing Diagnosis Association-International Nursing Intervention Classification, Nursing Outcomes Classification, and International Classification for Nursing Practice (ICNP) in the test section of the EHRs to gather nurses’ preferences. Quantitative data collection, focus groups, and survey analyses were conducted.

**Results:**

Nurses chose the ICNP for its flexibility but sought better methods to define patient severity in assessments and outcomes. They suggested incorporating the Common Terminology Criteria for Adverse Events to enable context-sensitive care plans.

**Conclusions:**

End-user involvement is essential for developing EHRs, enhancing system usability, and reducing implementation resistance. Including nurses in management decisions empowers them, and improves care quality.

Nurses employ care plans to implement the nursing process, which consists of defined and scientifically validated nursing diagnoses, interventions, and predicted patient outcomes. These components effectively describe patients’ conditions and how nurses deliver care. It is also widely acknowledged that the adoption of standardized nursing terminology (SNT) enhances communication among nurses through the use of shared lexicon definitions, promotes critical thinking, and facilitates the exchange of clinical data.^1-4^ Therefore, SNTs represent an optimal approach for documenting advanced nursing processes in electronic health record (EHR) systems,^5^ ensuring patient safety and the quality of clinical data through the use of a unique, clear, and shareable language.^6,7^ Nevertheless, a key factor in effectively implementing an EHR system is its alignment with the specific context,^8^ necessitating the development of nursing documentation suited to the environment’s needs and complexities.^9^ This adaptation is equally important when incorporating SNTs into EHRs to produce consistent clinical data,^10^ as various nursing taxonomies exist, and multiple studies have evaluated SNTs for implementing nursing care plans in EHR systems.^7^

In line with the World Health Organization guidelines, in 2012 Italy legislated the digitalization of health care documentation, allowing hospitals to initiate the dematerialization process.^11^ The introduction of EHRs remains a major objective, although implementation across the country is still inconsistent. This issue primarily stems from differences in regional healthcare systems and local investment availability, which affect compliance with the Italian EHR laws. Furthermore, recent legislation includes language standardization in EHR systems to enhance data exchange.^12^ This likely deter hospitals from adopting EHRs and complicates the fundamental transformation of healthcare documentation. One of the first EHR systems was developed at our cancer center, which is classified as a research facility in Northern Italy. The hospital’s administration and the information and communication technology (ICT) department conducted a strategic assessment to evaluate readiness for EHR adoption, aiming to anticipate and mitigate potential user resistance, following the launch of several e-health initiatives since 2016. This involved reviewing paper-based nursing documentation and selecting an oncological nursing SNT to create care plans because of the absence of similar examples in the given context.

## Nursing Standardized Terminologies for EHR Systems

Currently, there is no specific oncological nursing taxonomy. Therefore, we considered 2 SNTs retrieved from the literature for the implementation of EHR care plans: the North American Nursing Diagnosis Association-International (NANDA-I) and the International Classification for Nursing Practice (ICNP).^13,14^

NANDA-I was created to classify nursing-related health care issues, offering a list of defining characteristics (signs and symptoms) and associated etiologic factors.^15^ The taxonomy also contains a list of evidence-based defined risk diagnoses and risk factors. It is often combined with the Nursing Intervention Classification (NIC) and Nursing Outcomes Classification (NOC) to provide a research-based taxonomy that encompasses standardized nursing diagnoses, interventions, and outcomes on a global scale.^16,17^ These combinations, known as the NANDA-I, NIC, and NOC taxonomy (NNN), provide terms for nursing judgments, interventions, and nursing-sensitive patient outcomes.^18^ The electronic version of NANDA-I is accessible through licensure, and the NIC and NOC have a distinct licensure from NANDA-I.^15^

The ICNP, an internationally recognized nursing taxonomy, categorizes nursing practices and bridges different nomenclatures through cross-mapping functions.^19^ The ICNP incorporates predetermined terminology for nursing diagnoses and interventions while also permitting the creation of specialized catalogs or nursing data subsets tailored to contexts through the use of a 7-axis model (focus, judgment, means, action, time, location, and client).^1(p381)^ Recently, the International Council of Nurses (ICN) Board signed an agreement with the Systematized Nomenclature of Medicine International (SNOMED International) to integrate ICNP content into SNOMED-Clinical Terms (SNOMED-CT), which consists of comprehensive health care terminology, and the ICNP SNOMED-CT Nursing Practice Refset was released. This agreement allows to use both ICNP and SNOMED-CT classifications together in SNOMED International Member countries.^20^

Research emphasizes the importance of involving users in EHR implementation.^21^ Consequently, the hospital administration launched the EHR project with end-user involvement from the outset. Likewise, when incorporating the nursing process into the EHR, the healthcare professional leadership team employed this strategy to develop the nursing documentation section, covering the complete nursing process.

## Purpose

The present paper describes a pilot project aimed at choosing an SNT and initiating the implementation of nursing care plans in the EHR of a Cancer Center classified as a research facility in Northern Italy.

## Methods

### Study Design

**Figure 1.**
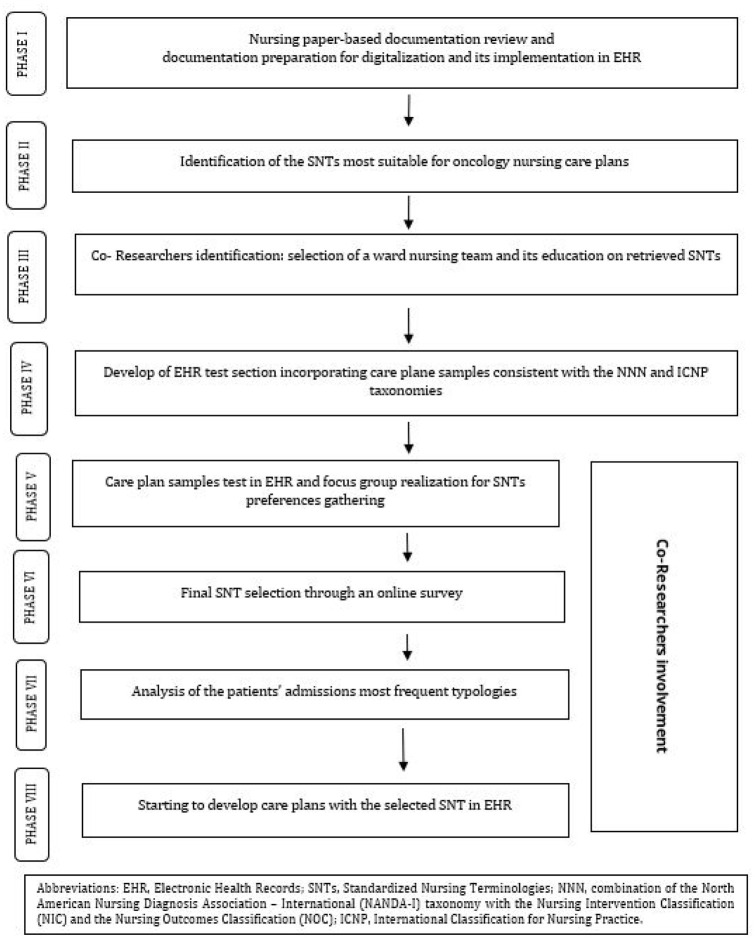
Study phases flow diagram.

The methodology of participatory action research (PAR) was chosen for its proven effectiveness, enabling researchers to engage directly with the study area and enhance nursing practice.^22^ PAR entails collaboration between researchers and participants to identify issues, choose methods, analyze data, implement findings, and enhance self-esteem, motivation, community solidarity, and awareness.^23^ In addition, PAR methods enhance health service users’ involvement in developing e-Health resources, thereby increasing their relevance.^24^ The PAR process was conducted from 2018 to early 2022 in a 415-bed cancer center certified by the Organization of the European Cancer Institute and is currently considered a national oncological referral center. This study followed current guidelines to ensure quality and transparency in reporting PAR in health research.^25^ The research process is illustrated in Figure 1.

The leadership team of health care professionals and a nursing educator from a bachelor’s School of Nursing collaborated to conduct this research. Their roles were: (1) reviewing nursing documentation and implementing the EHR, (2) analyzing literature for selecting a taxonomy suitable to oncologic settings, (3) training coresearchers on nursing taxonomies and nursing processes, (4) conducting focus groups to explore team preferences and gather opinions on the proposed care plans and SNTs, and (5) combining taxonomies with admissions typology classifications.

The nursing team of the hematology and medical oncology ward was chosen to play the active role of coresearchers in this project. This team (including the nurse in charge) was involved in the following 4 steps: (1) choosing an SNT suitable for the oncology setting; (2) identifying a set of nursing diagnoses, interventions, and outcomes to test the new EHR format; (3) creating a classification of hospitalization typologies; and (4) developing care plans suitable for the clinical setting.

In 2018, the leadership team of healthcare professionals and a nursing educator worked together to digitize and streamline paper-based nursing documentation, thereby creating an EHR nursing assessment section. A narrative literature review was conducted using major biomedical databases to identify examples of nursing care plans utilizing SNTs in oncology, guided by the clinical question: “What are the most frequent nursing diagnoses in the oncology setting?” The search was conducted in February 2018. Table 1 presents the search strategy. Two nursing classifications were retrieved and compared using Müller-Staub et al.’s^26^ evaluation criteria: NANDA-I combined with NIC and NOC and the ICNP classification.

**Table 1.** Biomedical Databases Search Strategy.

Databases	Research strategy	Records
PUBMED	((most common) AND (nursing diagnosis)) AND (oncology) Filters: Free full text, from 2013 to 2018	98
PUBMED	((NURSING DIAGNOSIS)) AND (ONCOLOGY)“from 2013 to 2018,”(((((““standardized nursing terminology”“[MeSH Terms] OR ((““standardized”“[ALL Fields] AND ““nursing”“[ALL Fields]) AND ““terminology”“[ALL Fields])) OR ““standardized nursing terminology”“[All Fields]) OR (““nanda”“[ALL Fields] AND ““international”“[ALL Fields])) OR ““nanda international”“[ALL Fields]) AND ((““nursing diagnosis”“[MeSH Terms] OR (““nursing”“[ALL Fields] AND ““diagnosis”“[ALL Fields])) OR ““nursing diagnosis”“[ALL Fields])) AND (((““neoplasms”“[MeSH Terms] OR ““neoplasms”“[ALL Fields]) OR ““oncology”“[ALL Fields]) OR ““oncology s”“[ALL Fields])”	3
CINAHL	Tl nursing AND Tl diagnosis AND Tl oncology	2
SCOPUS	Title (nursing AND diagnosis AND oncology) and limit-to (subj. “nurs”)	0

Abbreviation: nanda: North American Nursing Diagnosis Association.

Subsequently, the nurse educator trained the co-researcher nursing team on both SNTs through frontal interactive lessons. This training equipped the coresearchers for the next phase, creating a trial EHR section with 4 sample care plans using the NNN and ICNP taxonomies. These samples were created through cross-mapping^27^: SNT terms were compared with common nursing problems at the cancer center. Nursing care plans for diarrhea, nausea, hyperthermia, and chronic pain diagnoses were formulated. This section enabled care plans’ completion via predefined options to aid nurses’ choices, featuring 3 drop-down menus for taxonomy, diagnosis, and nursing outcomes. Two technical decisions ensured uniform completion and interface. As the ICNP does not include categorization for nursing interventions, the task did not require the integration of the NIC taxonomy of the NNN taxonomy. Consequently, 2 lists of nursing interventions with radio buttons that allowed multiple choices were created: one for the NNN taxonomy and the other for the ICNP classification. The second decision was to establish a unique outcome indicator suitable for both taxonomies, as the NOC taxonomy incorporates a different label for each outcome indicator, whereas the ICNP taxonomy does not incorporate any outcome indicators. Thus, another drop-down menu was added, allowing the choice of one outcome-level indicator from the 5 available options: (1) severe, (2) substantial, (3) moderate, (4) mild, and (5) none. Time- and bar-colored alert systems were also associated with each outcome-level indicator on the EHR test home screen in which all patients in the ward were listed. For Level 1 and 2 outcome indicators, a 2-h time alert was established, accompanied by a yellow bar, which would turn red if nurses failed to do action within another hour to attract their attention and prompt action. For Level 3 outcome indicators, a deadline of 12 h was set, and the yellow bar turned red if nurses did not act within another hour. If nurses were assigned an outcome indicator of Level 4, a 24-h alert was associated with a green bar, which would turn yellow if no action was taken after the time limit, and red if no action was taken within another hour, indicating the need for urgent intervention. With an outcome indicator of Level 5, the associated bar was green, indicating that the healthcare problem was solved. A nurse line manager oversaw the EHR implementation, working with the ICT department and the EHR vendor. A practice supply manual for the EHR test section was developed to support the trial. Beginning on December 23, 2019, the co-researcher team tested the sample care plans.

The project was interrupted in February 2020 owing to the COVID-19 pandemic, resumed in June 2020, and halted again in October 2020. During this time, adjustments were made to the EHR trial section based on the nursing team’s observations and suggestions.

### Data Collection

An ICT member extracted data from the EHR test section for frequency analysis of the care samples tried by the nursing team. The number of nursing diagnoses, interventions, and outcomes selected from the ICNP and NNN taxonomies were extracted and used to create pivot tables in Microsoft Excel Professional Plus 2019 (Microsoft Corp., Redmond, WA, USA). The data were then sorted in descending order according to the frequency with which the nurses selected them.

In the first trimester of 2021, consistent with previous research,^28^ a 2-round focus group was conducted following the Consolidated criteria for Reporting Qualitative research (COREQ).^29^ The interviews sought to investigate coresearchers’ preferences and opinions on the proposed care plans and SNTs. A researcher, supported by the nurse ward manager, led the group, clarified the study aims, verified voluntary participation, and obtained written consent for the audio recording. The focus groups were held in person and partially via conference calls in a private room. The discussions were guided by the following questions: (1) Did you use care plan samples? If so, please explain your reasoning below. If not, please explain the reason. (2) Which of the proposed taxonomies do you think best fits nursing planning in our oncology settings? (3) What modifications would you suggest enhancing the functionality of the EHR nursing planning section and effectively attain the objective of presenting nursing interventions aimed at addressing patients’ care issues? (4) What are the educational requirements for using the EHR trial section?

The first focus group on March 23, 2021 lasted 1.5 h with 10 nurses participating. The second, on March 24, 2021, also lasted 1.5 h, with 8 nurses attending both in person and remotely. Focus-group discussions were transcribed verbatim, and each transcript was verified for accuracy before content analysis.^30^ The concepts derived from the interviews were grouped into categories preparing for their abstraction and consequent interpretation.^31^ The themes identified through focus-group content analysis guided the subsequent pilot study phase in April 2021.

This phase involved surveying the selected nursing team to determine a definitive decision between the ICNP and NNN taxonomies. The survey comprised 2 phases. First, a sample care plan for hyperthermia diagnosis was introduced, developed using both classifications and detailed within the same framework as the EHR test section, incorporating focus-group modifications. The predominant taxonomy was designated as the preferred SNT when data extraction from the EHR was conducted for the frequency analysis. Subsequently, an online survey using Google Forms was executed, consisting of 2 closed and 2 open-ended questions: (1) Which of the suggested taxonomies in the 2 samples best describes nursing interventions for the proposed problem? (2) Provide a detailed description of the motivation for the choice; (3) In relation to the choice, could you propose modifications to simplify the process or deepen the content? (4) If that’s the case, please note the required modifications. The survey analysis included extracting response frequencies for 2 closed-ended questions, ordering them by selection, and performing a content analysis on the 2 open-ended questions.

Subsequently, in May 2021, within the selected ward, the patient’s case mix and complexity were assessed by initially analyzing the reasons for their admissions. This procedure, developed by the coresearchers, stemmed from focus-group analysis, indicating that care plans should more accurately reflect patients’ true complexity. Thus, as the hematology and medical oncology ward focuses primarily on hematological malignancies and conducts trials on solid cancers, the most frequently used medical treatments and chemotherapy regimens were selected to classify admission typologies. A comprehensive list of the most common healthcare issues was created for each admission typology using scientific language instead of specialized nursing terminology. Completed in the first 2 weeks of June 2021, this preparatory work laid the groundwork for translating each identified healthcare issue into the SNT chosen via the survey.

Consequently, 2 work groups were formed. One aimed to link common healthcare issues with admission types to the Common Terminology Criteria for Adverse Events (CTCAE) classification,^32^ version 5.

The US National Cancer Institute developed the CTCAE, comprising standardized terms for describing adverse drug effects in cancer therapy. Each term is graded from 1 to 5, indicating severity. Grade 1 refers to asymptomatic or mild symptoms; grade 2, moderate symptoms; grade 3, severe or medically significant symptoms; grade 4, life-threatening symptoms; and grade 5, death due to adverse effects.

The second workgroup focused on the healthcare issues, diagnoses, interventions, and outcomes of the selected SNT. Specific oncological contexts sometimes complicate identifying SNT diagnoses. Analyzing healthcare terminology definitions helped formulate accurate diagnoses.

The first issue addressed was mucositis, as suggested by coresearchers, which was evaluated primarily using CTCAE. The most suitable ICNP diagnosis was selected based on the CTCAE score for mucositis, followed by the selection of appropriate interventions and outcomes. A new EHR section, *Nursing Planning*, was launched on January 3, 2022, with coresearchers starting its use. Additional healthcare issues were mapped to the CTCAE and ICNP classifications to develop comprehensive inpatient care plans for the hematology and medical oncology wards. Several audits of the EHR nursing care plan documentation were conducted, involving coresearchers to review and suggest improvements. This ongoing methodology allows for the gradual incorporation of all care plans developed for patients in this pilot project ward.

### Ethical Considerations

The Internal Scientific Board approved the project via e-mail on April 26, 2018. The nurses in our pilot study consented to sign a standard institutional form used in clinical research. Ethical permission was not required, as this pilot study aimed to develop nursing care plans for EHRs in a single ward and its staff. The principles of the Declaration of Helsinki have been upheld.

## Results

The coresearchers consisted of 18 nurses, averaging 35 years of age, with working experience in the cancer center ranging from 2 months (2 nurses) to 34 years (the nurse in charge). Of these nurses, 72% (*n* = 13) held a bachelor’s degree in nursing and 58% (*n* = 10) had a postgraduate specialist course (60 ECTS credits). The care plan samples in the EHR test section were implemented in 57 patients, with 156 applications. Nurses selected the ICNP taxonomy in 54% of the cases (85 times) and the NNN taxonomy in 46% (71 times). Table 2 summarizes the most common diagnoses, planned outcomes, and corresponding interventions for each taxonomy, in which the data showed consistency among the chosen diagnoses, planned outcomes, and nursing interventions. For instance, the most commonly chosen interventions for the ICNP outcomes “No Pain” and “Pain Control” included “Teaching about managing pain,” “Assessing pain control,” “Monitoring pain,” and “Administering pain medication.” The selection of the NIC “Nausea and Vomiting Control” corresponds to the NOC categories “Nausea and Vomiting Control,” “Pain Level,” “Symptom Control,” and “Nutrition Status.”

**Table 2.** Summary of Taxonomy Data in the EHR Test Section.

Category	ICN taxonomy	NNN taxonomy
Frequency of selection	54% of care plans (85 out of 156)	46% of care plans (71 out of 156)
Most frequent diagnosis	- Chronic pain: 48% (*n* = 40) - Nausea: 37% (*n* = 31)	- Chronic pain: 54% (*n* = 38) - Nausea: 37% (*n* = 26)
Planned outcomes	- Pain control - No nausea	- Nausea and vomiting control - Pain level, pain control
Selected interventions	- Teaching about managing pain - Assessing pain control - Monitoring pain - Administering pain medication - Monitoring nausea - Administering antiemetic medication - Educating on diet and nutrition	**-** Pain assessment and education - Monitoring pain - Administering analgesics - Educating on managing nausea - Monitoring nausea - Nutrition and hydration support

Abbreviations: EHR: electronic health record; ICN, International Council of Nurses; NANDA-I: North American Nursing Diagnosis Association-International; NIC: Nursing Intervention Classification; NNN: NANDA-I, NIC, and NOC taxonomy; NOC: Nursing Outcomes Classification.

During the focus-group discussions, 4 main issues regarding the EHR emerged, as presented in Table 3. Coresearchers primarily emphasized revising the EHR to accurately represent the nursing process, including assessment, diagnosis, and outcome evaluation. Focus-group analysis revealed nurses’ willingness to improve electronic systems by proposing changes, such as home screen alerts, to quickly address discrepancies identified by outcome indicators. The analysis highlighted their active participation in the EHR care plans project, recognizing the importance of electronic nursing documentation and supporting an enhanced informatics system.

**Table 3.** Categorization of Focus-Group Interviews’ Emergent Content.

Features of the EHR trail module	• Provides little information • Confused • Not focused • Not clearly visible within the EHR • The selected interventions not able to clearly address the patients’ problems
Implications of the EHR module on nursing activity	• Time-consuming, but it could be helpful if simplified
Emergent problems during the test	• Computer problems, such as login mode
Proposal of EHR module adjustments	• Develop a grading system for healthcare problems • Create a ward manual that includes nursing planning instructions for the most common ward healthcare problems • Incorporate a discharge section that includes care plans with relevant information • Develop a nursing planning section with predefined profiles for each type of healthcare problem • Provide options for different planning time frames (short, medium, and long) • Add interventions related to transfusions and blood cultures

Abbreviation: EHR: electronic health record.

A preference for the ICNP taxonomy has emerged from the care plans test for the hyperthermia diagnosis, in line with the successive survey findings, administered to 18 nurses, and 14 responses were received. Of the respondents, 57.1% (*n* = 8) confirmed their preference for the ICNP taxonomy. They motivated their choice, affirming that they found the ICNP taxonomy more synthetic, but immediate, clear, and easy to employ in practice. Coresearchers who favored the NNN taxonomy asserted that it permits the evaluation of healthcare problems from different perspectives and that the NOC is more complete in describing nursing outcomes. The survey also revealed that most nurses asserted the need to add more detail, regardless of their preferred taxonomy. The surveyed nurses recommended linking a grading system to both taxonomies to more accurately assess the severity of healthcare issues and formulate care plans. They recommended integrating nursing care plans with the CTCAE system, which is routinely used by oncologists to evaluate the side effects of chemotherapy. The suggested integration of the CTCAE into nursing care plans was favorably evaluated by the researchers.

In the successive analysis of clinical activities in the hematology and medical oncology ward, 4 primary categories of patient admission were identified: (1) cancer treatment, including chemotherapy and conditioning regimens prior to allogeneic hematopoietic stem cell transplantation; (2) supportive therapies for managing chemotherapy side effects or cancer progression; (3) post-diagnostic and staging treatment planning; and (4) administration and monitoring of Phase I trial treatments or bispecific antibodies. Each admission category for health care issues was cross-mapped with the corresponding ICNP diagnoses and selected CTCAE terms.

## Discussion

This pilot study aimed to start the introduction of nursing care plans developed using an SNT in the EHR of a cancer center, following an initial EHR implementation phase. Following the initial EHR implementation phase, healthcare professionals’ compliance was evaluated and the results were integrated into the current project. Despite administrative readiness for a new EHR project, professionals were wary of past experiences and had limited involvement as end users. These factors and previous research on transitioning to electronic documentation were considered^33^ and the hospital administration assembled a multidisciplinary team consisting of healthcare professionals, the EHR project manager from the ICT department, and the vendor. This strategy aligns with research emphasizing the vital role of strong leadership in EHR transitions, which is crucial for preventing medical errors, improving clinician communication, and enhancing care quality.^8^ The team incorporated colleagues’ suggestions to customize the EHR system for healthcare professionals and develop unique components such as nursing care plans, which were previously unavailable locally.

These considerations guided the selection of wards and nursing teams, ensuring hospital representation and alignment with the EHR study outcomes. The first aspect considered was linked to the patient case mix in the hematology and medical oncology ward, providing an accurate representation of the complexity of nursing care at the Oncology Center. Second, the ward manager’s leadership and nurses’ willingness to engage in teamwork were consistently recognized. This choice aligns with studies indicating that selecting a clinical sample and its active involvement can effectively reduce resistance during EHR implementation, suggesting the appropriateness of the PAR methodology for this study.^9,24,28^ Furthermore, the nurse educator involved in this project had previously worked in that ward. This past experience enabled collaboration between EHR developers and end users, which a recent systematic review confirmed as essential for successful EHR implementation.^34^ The nursing team was also selected based on their confidence in ICT systems and a minimum required knowledge of SNTs, as recommended by the literature.^24,25^ Nurses in this project fully explored ICT applications, maximizing their potential and actively contributing to improvements for future users. They functioned as superusers, resolving issues across the computer network, EHR, and other technologies, thereby supporting EHR implementation and aiding future user compliance, as noted in the literature on electronic clinical documentation implementation.^34-36^

The creation of an EHR test section enabled customization of the application, as proposed in the reviewed literature, to improve accessibility, clarity of content, and efficiency of completion.^6,23,25^ A few technical issues related to the testing of the EHR care plan samples were identified and resolved. One issue was the lack of an alert system linked to outcome indicator grading. Adding this system facilitated nurses’ actions by setting intervention expiration times. Coresearchers positively evaluated the practicality of the outcome indicators on the EHR test home screen, allowing quick access to care plans and required actions. These outcomes are consistent with the findings of McBride et al, who reported in their study that EHR alerts to facilitate adherence to clinical standards were well received by nursing staff.^37^

In testing the care plan samples, coresearchers also stated that both taxonomies were acceptable for representing the nursing process in the specific setting of the Cancer Center. The survey indicated that the ICNP was more practical and adaptable. Although nurses were more familiar with NNN, which is commonly taught in Italian nursing schools, linking NIC and NOC with recurring nursing issues in oncology was difficult. Unlike NNN, ICNP diagnoses do not need defining characteristics and related factors (etiology).^1^ The ICNP enables the customization of nursing terminology through pre-coordinated concepts for diagnoses, outcomes, and interventions (outcomes diagnosis and interventions), and by combining concepts from the 7 axes.^38^ It is probable that nurses considered this aspect an advantage in terms of simplicity rather than a critical issue in ICNP terminology. The internationally recognized NNN and ICNP nursing languages consistently define advanced nursing care plans in both paper and electronic formats, as confirmed by multiple studies.^7^ However, the intrinsic features of ICNP allow for setting-specific nursing diagnoses and interventions. This process starts by analyzing healthcare problem characteristics to identify the most appropriate taxonomy concepts, which are then combined to create a consistent nursing diagnosis.

SNTs are crucial for defining, explaining, and understanding nursing practice. Nevertheless, nurses working in an interdisciplinary context acknowledge the importance of facilitating communication between various disciplines. Coresearchers have requested a grading system for both ICNP and NNN taxonomies to more accurately define patient condition severity during assessment and outcomes evaluation. They promptly suggested the implementation of the CTCAE score system as the medical staff consistently utilized it for clinical purposes as well as multidisciplinary communication. This underscores the necessity perceived by healthcare professionals to adopt shareable communication means and terminologies to enhance patient information exchanges and, consequently, advance the standard of care.^39^ The integration of CTCAE with the ICNP taxonomy was a critical factor that persuaded the coresearchers regarding the reliability of the project. The team viewed the inclusion of the CTCAE as a natural and practical complement to the chosen nursing taxonomy. Consequently, the combination of ICNP and CTCAE facilitated the development of care plans.

### Implications

Despite the prevalent use of NNN taxonomy, coresearchers favored ICNP, which is already cross-mapped with SNOMED-CT. This classification provides common terms for consistently defining different and specific practices of separate clinical disciplines and therefore connects disparate existing taxonomies.^10(p8),40^ The unexpected preference for ICNP could impact EHR data-mining processes vital for research,^39^ quality care, and standards improvement, as Italy already integrates the SNOMED-CT taxonomy, being a SNOMED International country member. Our empirical observations suggest a wider adoption of the ICNP classification in the EHR system to explore its potential for enhancing nursing practice and research. In addition, evaluating the informative capacity of data extraction using the SNOMED-CT combination would be advantageous.

The development of an EHR presents a daunting challenge, as previous studies have shown. If end users are not effectively engaged, they may resist implementation, perceiving the EHR as an obstacle to their normal workflow, rather than as a beneficial instrument.^41^ The focus group and care plan test enabled the exploration of EHR usability and usefulness issues and the consequent effort to implement technological improvements to avoid workarounds that could challenge patient safety.^35^ However, as the literature is aware, EHR systems are often highly contextualized to ensure customization, potentially affecting the generation of reusable data for quality improvements and research purposes.^42^ In our experience, involving end users as coresearchers throughout the project posed challenges, occasionally necessitating mediation between researchers prioritizing strict adherence to the nursing process and the nursing team requiring contextualization to effectively address patients’ needs. Therefore, it is important to develop EHR systems that incorporate structured data as terminology classifications that can yield.

### Limitations

This project aimed to implement nursing care plans using a specific nursing taxonomy in an institutional EHR. The goal was to create a tool that accommodates patients’ complexity and specific context needs, balancing customization with the generation of reusable data for future research and quality care enhancement. Thus, the strategies and methodologies adopted for implementation cannot be generalized. The adoption of CTCAE in combination with ICNP taxonomy should not be interpreted as an ICNP modification but rather as an experimental attempt to integrate multidisciplinary classification systems. The initial section of the EHR test presented significant technical challenges that were further compounded by the slowdown caused by the pandemic, which caused a delay of several months in all phases of the project.

## Conclusions

Introducing an EHR test section for nursing process implementation revealed that the ICNP taxonomy was more adaptable to the cancer center’s context and complexity. However, neither taxonomy addresses cancer-specific settings. This project can initiate integrating SNTs into oncological contexts and defining a subset of concepts linked to oncological health care issues. The ICNP exhibited the necessary flexibility to initiate the process, allowing for the free construction of nursing terminology and the creation of pre-coordinated concept catalogs. This project enabled the establishment of a structured nursing plan section within the institutional EHR for the cancer center. Future initiatives could involve implementing care plans for other nursing diagnoses and extending the project to different clinical case mixes to standardize the communication of care issues in hospitals. Evaluating the feasibility of data mining through ICNP and SNOMED-CT linkages would also be valuable for extracting real-world data, contributing to advancements in nursing practice and research.

Identifying a nursing team as both coresearchers and “superusers” was crucial, as they customized the EHR section and made the system user-friendly according to the literature.^39^ This study highlights the importance of involving end users in EHR implementation to ensure proper usage, patient safety, and accurate healthcare documentation. Efforts to improve the design and functionality of the Nursing Plan section in EHR are ongoing.
